# A New Current-Shaping Technique Based on a Feedback Injection Mechanism to Reduce VCO Phase Noise

**DOI:** 10.3390/s21196583

**Published:** 2021-10-01

**Authors:** Francisco Javier del Pino Suárez, Sunil Lalchand Khemchandani

**Affiliations:** Departamento de Ingeniería Electrónica y Automática, Institute for Applied Microelectronics (IUMA), Universidad de Las Palmas de Gran Canaria, E-35017 Las Palmas de Gran Canaria, Spain; jpino@iuma.ulpgc.es

**Keywords:** LC-VCO, CMOS, phase noise, current-shaping, 90 nm, current tail, varactor, LC tank, on-wafer

## Abstract

Inductor-capacitor voltage controlled oscillators (LC-VCOs) are the most common type of oscillator used in sensors systems, such as transceivers for wireless sensor networks (WSNs), VCO-based reading circuits, VCO-based radar sensors, etc. This work presents a technique to reduce the LC-VCOs phase noise using a new current-shaping method based on a feedback injection mechanism with only two additional transistors. This technique consists of keeping the negative resistance seen from LC tank constant throughout the oscillation cycle, achieving a significant phase noise reduction with a very low area increase. To test this method an LC-VCO was designed, fabricated and measured on a wafer using 90 nm CMOS technology with 1.2 V supply voltage. The oscillator outputs were buffered using source followers to provide additional isolation from load variations and to boost the output power. The tank was tuned to 1.8 GHz, comprising two 1.15 nH with 1.5 turns inductors with a quality factor (Q) of 14, a 3.27 pF metal-oxide-metal capacitor, and two varactors. The measured phase noise was −112 dBc/Hz at 1 MHz offset. Including the pads, the chip area is 750 × 850 μm${}^{2}$.

## 1. Introduction

Voltage-controlled oscillators (VCOs) are widely used in the design of sensor systems. VCOs are generally found in transceivers for ultra low-power wireless sensor networks (WSNs) where, in conjunction with the phase locked loop (PLL), are used for frequency synthesis, fast switching circuits, and clock recovery [1,2,3,4,5,6,7,8,9,10]. VCOs are also a fundamental part of VCO-based reading circuits where the sensor core output voltage is applied to the VCO tuning voltage node, achieving high sensitivity and high signal-to-noise ratio compared to amplifier-based reading circuits [11,12,13,14]. Also, distance, speed and other parameters can be remotely measured in real time using VCO-based radar sensors that monitor the electromagnetic wave shift between transmitted and received signals [15,16,17]. The purity of the VCO output signal greatly influences the operation of these systems and, for this reason, the main considerations when designing a VCO are low phase noise, minimal chip area, low power dissipation, and high operating frequency. Today’s nanoscale complementary metal-oxide semiconductor (CMOS) technology can meet most of these requirements. However, reducing phase noise is still a major issue, mainly due to the poor performance of CMOS process in terms of flicker noise [10]. The major approach that has been used to reduce VCO flicker noise is to apply biasing techniques to both the VCO core transistors and the current source transistors needed to supply the DC bias current of the core transistors. In [18], a review of techniques for reducing CMOS VCO phase noise caused by flicker noise is presented. This study focuses on current source transistors biasing techniques and concludes that current-shaping techniques can significantly reduce its flicker noise contribution to the output phase noise.

In this paper a new current-shaping technique is proposed to reduce the VCO’s phase noise. The proposed technique is based on a feedback injection mechanism and only uses two additional transistors. Using this approach, a significant phase noise reduction is achieved with a very low area increase. Section 2 introduces the techniques for the reduction of the phase noise of CMOS based VCOs and describes the advantages of the current-shaping techniques. Section 3 presents the proposed topology and analysis, followed by the experimental results in Section 4. Finally, Section 5 concludes the paper.

## 2. Current-Shaping Biasing Techniques

**Figure 1:**
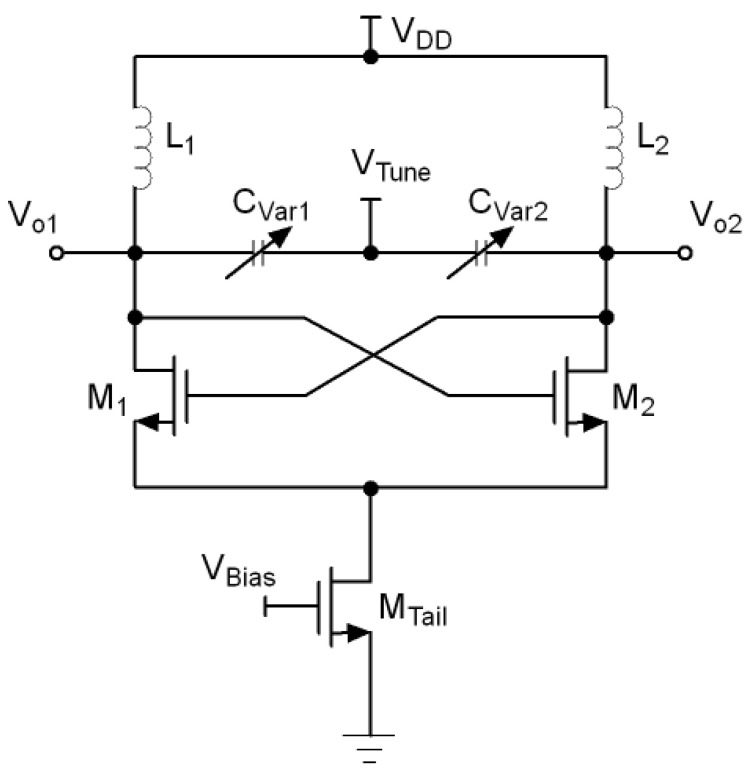
Conventional LC-VCO.

**Figure 2:**
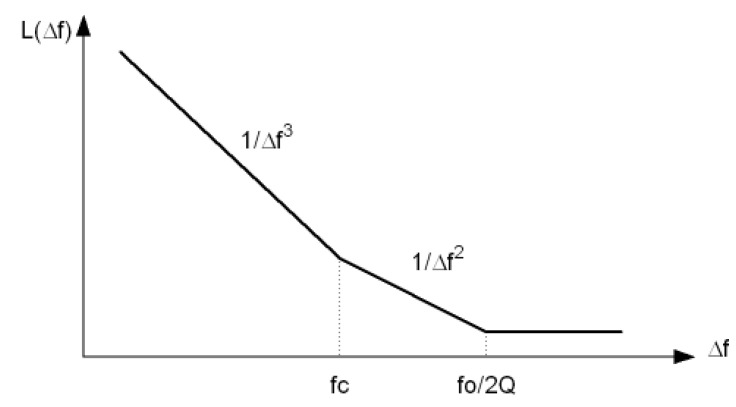
Phase noise vs. frequency.

One of the most used topologies for the implementation of VCO circuits is the inductor-capacitor voltage controlled oscillator (LC-VCO) since they show less phase noise although they occupy a high area due to the presence of inductors and dissipate more power. Figure 1 shows the conventional structure of an LC-VCO. The bulk of the NMOS transistors were connected to lowest potential which is ground. The close-in phase noise behaviour at an offset Δf from the carrier frequency $f_{0}$ is given by Leeson’s model [19].

(1) $$L\left(\Delta f\right)=\frac{1}{2}\frac{K\;T\;F}{P_{sig}}\left(1+\frac{f_{c}}{\Delta f}\right){\left(1+\frac{f_{0}}{2\;Q\;\Delta f}\right)}^{2},$$

where *K* is Boltzmann’s constant, *T* is the absolute temperature, *F* is the excess noise factor, $P_{sig}$ is the signal power, *Q* is the resonator loaded quality factor, and $f_{c}$ is the flicker noise corner where flicker noise and thermal noise are equal. This equation leads to the typical plot of phase noise versus offset frequency of Figure 2 and it also offers design insight on how to minimise the overall phase noise. It is well known that a lower excess noise factor (*F*), a larger amplitude of oscillation ($P_{sig}$), or a better tank quality factor (*Q*) results in an improved phase noise.

The previous analysis is based on a linear time invariant analysis of the oscillator. However, a more detailed analysis based on transient simulations indicates that the tail current bias noise can contribute strongly to the total phase noise [20]. The reason behind this phenomenon is that the switching transistors (M1 and M2) behave like an up-conversion mixer and convert the flicker noise of the tail current into AM noise at the output of the VCO which is subsequently converted into PM noise by the non-linear varactor. Also, the same mixing mechanism convert the tail current noise at the harmonics of the frequency of oscillation ($\omega_{0}$) directly to PM noise at the output through the indirect FM phenomenon [10,20].

**Figure 3:**
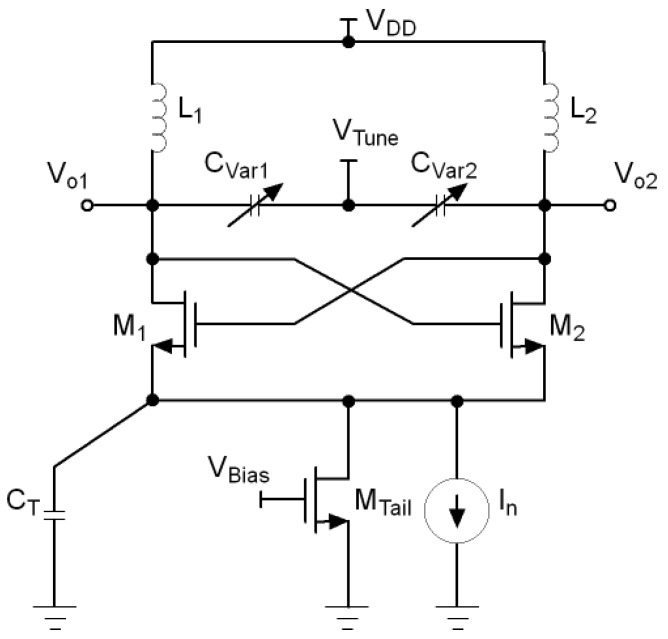
LC-VCO with tail current-shaping.

A possible solution to remove the harmonics of the tail current source is to filter them out by a capacitor, as shown in Figure 3 [21]. This technique is known as tail current-shaping and consist of reducing the tail current at the moments when the oscillator is most sensitive to noise, that is, when its effective impulse sensitivity function (ISF) is higher. The more symmetrical the shape of the tail current, the fewer harmonics it will have and therefore the lower the phase noise of the VCO.

The contribution of the tail current bias noise to the total phase noise is further aggravated if during the oscillation M1 and M2 enter the deep triode region. If this occurs, then two effects raise the phase noise. First, the on-resistance of M1 and M2 degrades the Q of the tank and second, the impulse response from the noise of both transistors contributes substantially to the output phase noise.

**Figure 4:**
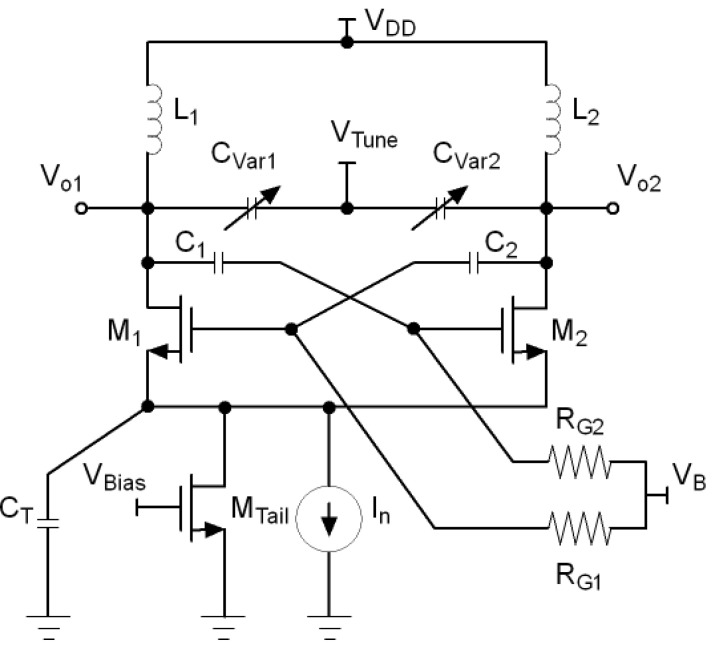
Class-C LC-VCO.

To avoid operation in the triode region but at the same time have large output swings, capacitive coupling can be inserted in the loop as shown in Figure 4 [22]. This topology is known as Class-C oscillator. In this topology, a bias voltage ($V_{B}$) is chosen so that M1 and M2 operate in the active region and, at the same time, allow a sufficiently high voltage swing at the drains to improve the phase noise performance. However, the peak output swing is limited by the tail capacitor and therefore, once a maximum is reached, no further improvement in phase noise can be achieved.

**Figure 5:**
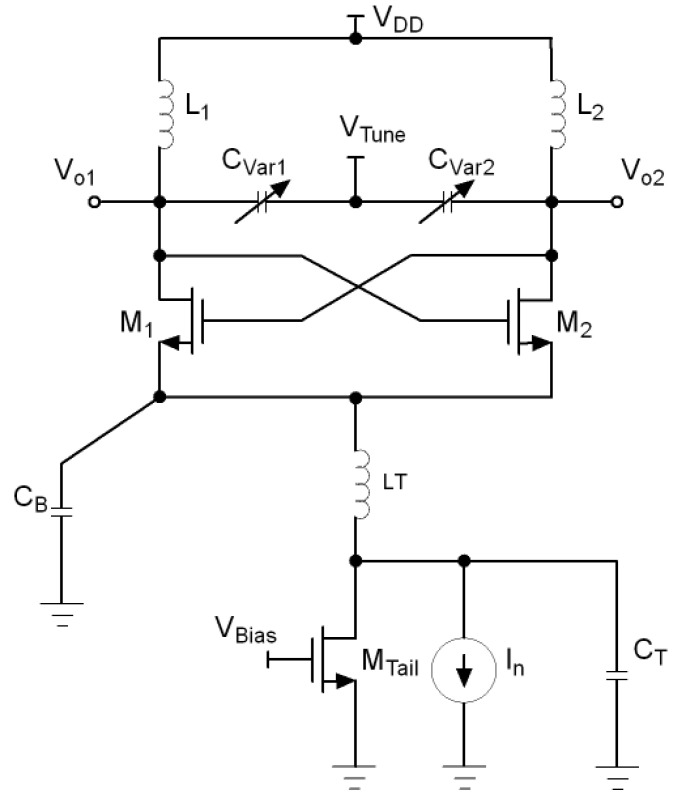
Filtering technique to lower LC-VCO phase noise.

Another approach is to allow the switching transistors to enter the triode region but eliminating the effect of the tail capacitance at $2\omega_{0}$ introducing an inductor in series with the tail node, as shown in Figure 5 [23]. The value of the inductor is chosen such that it resonates with the parasitic capacitance at the tail node at the second harmonic. With this topology larger swings can be achieved at the cost of a larger area due to the use of an additional inductor.

Noise cancellation topologies such as presented in [20,24,25] have also been employed to cancel the tail current noise component. However, they are hardly effective at high frequencies because they are able to cancel only the contribution of flicker noise, leaving the thermal noise unaffected.

**Figure 6:**
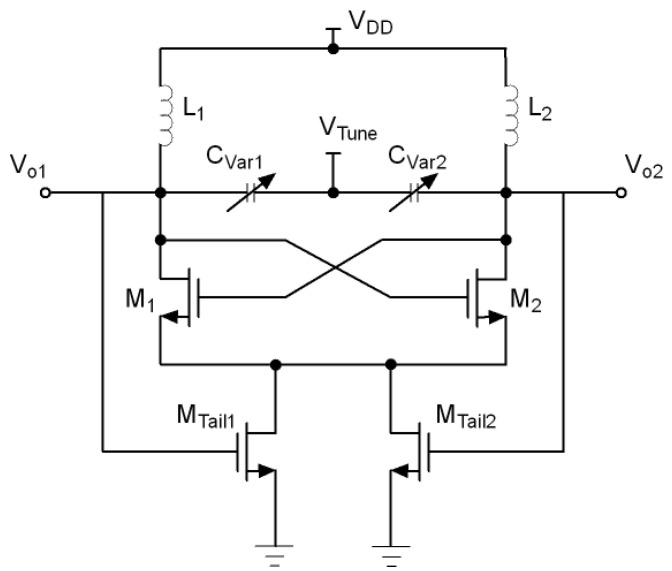
Tail current-shaping by coupling the VCO output voltage directly to the gates of two tail transistors.

**Figure 7:**
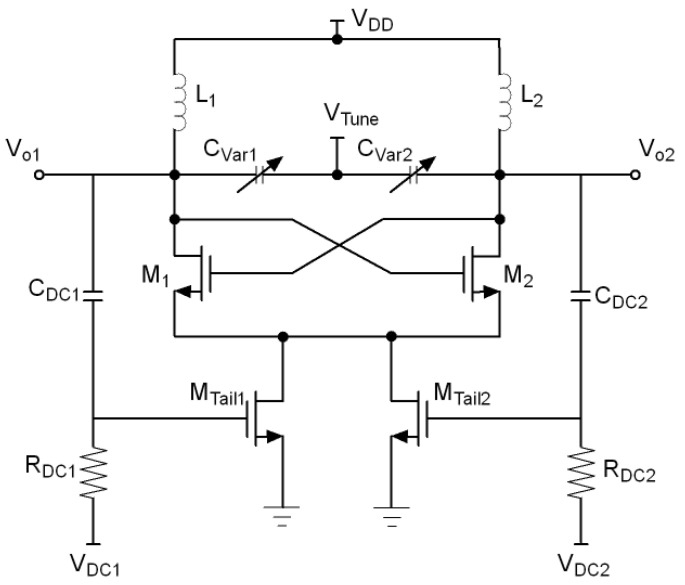
Tail current-shaping by coupling the VCO output voltage to the gates of two tail transistors decoupling the DC part from the AC part using a capacitor.

Many current-shaping techniques have been proposed in recent years to improve the VCO phase noise performance. One approach is the use of a switched biasing technique where an external pulse is injected into the gate of the tail current transistor [21]. This method has proven to reduce the phase noise, but its drawback is that an external pulse signal is required for locking. To avoid this, some authors have proposed to shape the tail current using the oscillator’s own output waveform as self-injection signal. A simple but high area cost method is to couple the oscillating signal VCO to the tail current source through a transformer [26]. Another approach is to couple the VCO output voltage directly to the gates of two tail transistors as shown in Figure 6 [27]. Although this method considerably reduces the area, the downside is that both, the AC and the DC parts of the output voltage are coupled to the tail transistors, resulting in high bias voltage of the tail current sources and, as a consequence, in high power consumption and flicker noise. One way to avoid this is to decouple the DC part from the AC part of the output voltage using a capacitor as shown in Figure 7 [28,29,30,31]. In this way, the DC part of the gate voltage of the tail transistors comes from an external source while the AC part comes from the VCO output. This allows to choose a DC voltage small enough so that the current supplied to the switching transistors is significantly reduced at the zero crossing points of the output, thereby reducing phase noise.

Feedback injection currents have also been proposed to improve the phase noise by modifying the triode region loading effect of the switch transistors and increasing the output transconductance. This method also causes a self-locking between the output voltage and currents at the zero crossing points, further reducing phase noise [32]. In this paper we propose a new current-shaping technique based on a feedback injection mechanism. The proposed topology significantly reduces phase noise by using only two additional transistors. In the next section we describe the proposed technique.

## 3. Proposed Topology

**Figure 8:**
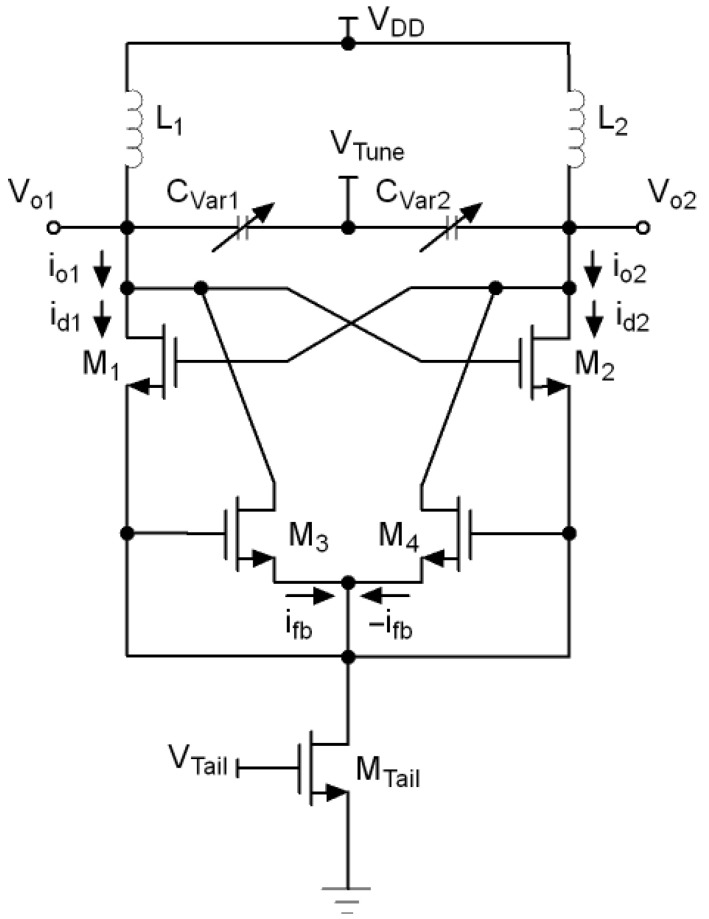
Proposed feedback injection mechanism to reduce the VCO’s phase noise.

The schematic of the proposed VCO is shown in Figure 8. The VCO core uses a cross-coupled transistor pair, M1 and M2, to build up the negative resistance. To ensure the loading effect and to improve the current feedback, $i_{fb}$ and $-i_{fb}$, the network composed by M3 and M4 is included.

In a conventional VCO, $V_{o1}$ and $V_{o2}$ voltages decrease and increase respectively during a half cycle of oscillation. If the cut-off regions are ignored, M1 and M2 swing between triode and saturation regions. For this reason, the drain-source resistances are not the same in both regions, being lower in triode. This increases the loading effect on the LC tank in this region, thus degrading the phase noise of the VCO.

In the proposed circuit, when $V_{o1}$ decreases and $V_{o2}$ increases, a feedback current $i_{fb}$ from node $V_{o2}$ is injected into node $V_{o1}$ through M4 and M3 transistors. This increases $i_{d1}$ and pulls M1 back from triode towards saturation region. The impedance seen from the LC tank towards the $V_{o1}$ node rises due to the virtual magnification of the drain-source resistance of M1. Then, the negative resistance seen from LC tank remains with the same value during the full oscillation cycle. The same explanation is applied to M2.

**Figure 9:**
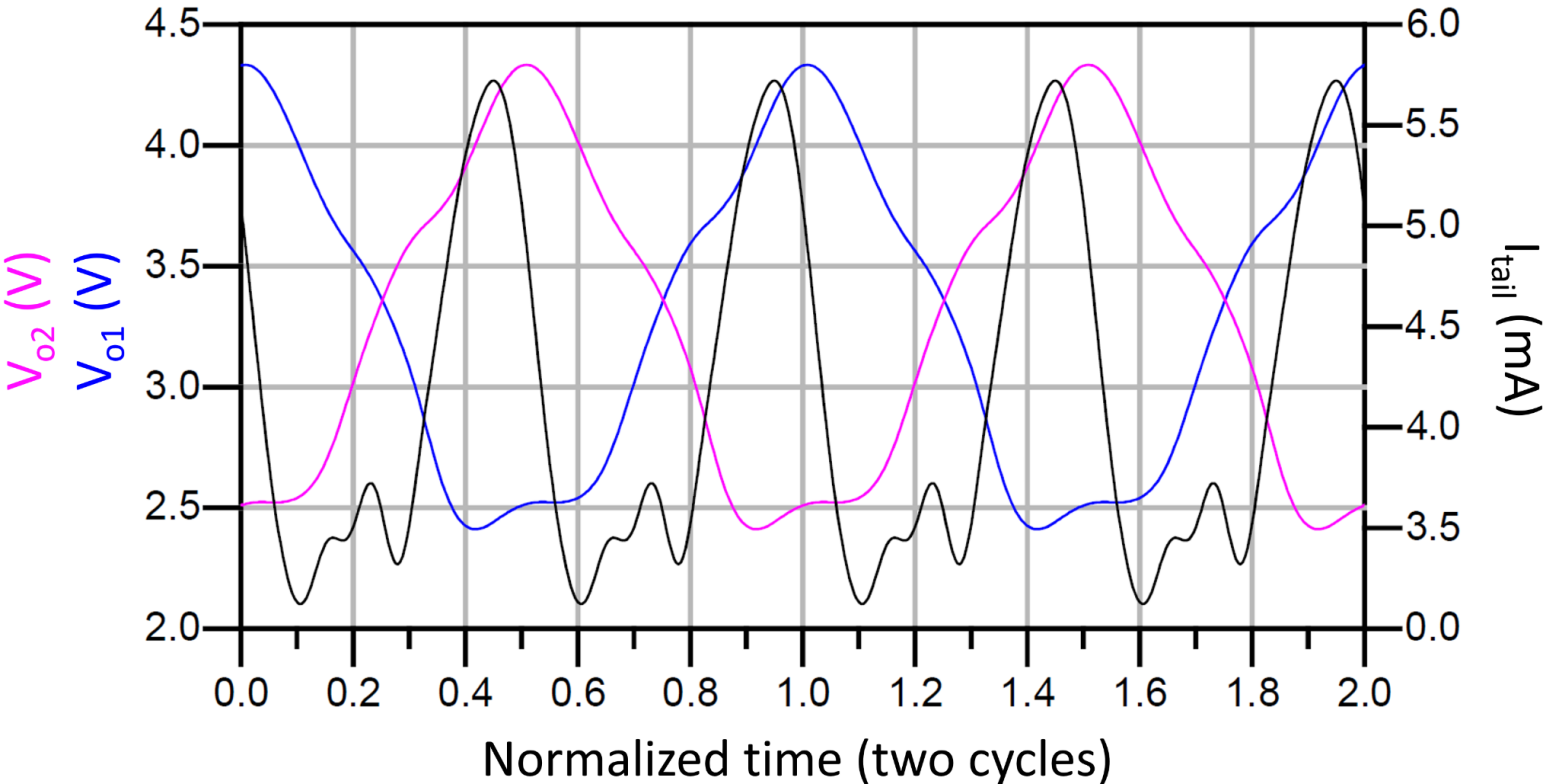
$V_{o1}$ and $V_{o2}$ and $I_{tail}$ of the conventional VCO.

**Figure 10:**
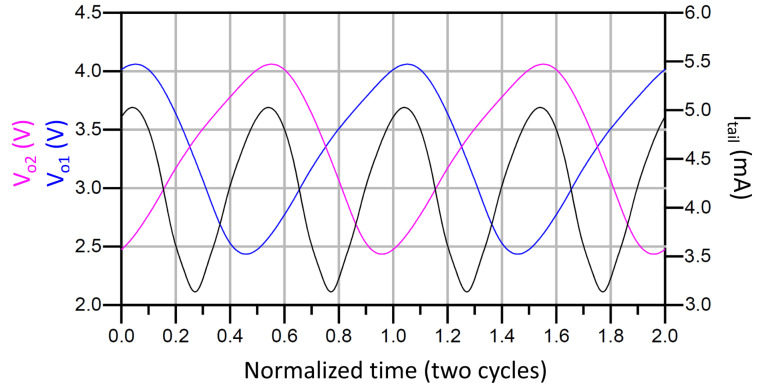
$V_{o1}$ and $V_{o2}$ and $I_{tail}$ of the proposed VCO.

**Figure 11:**
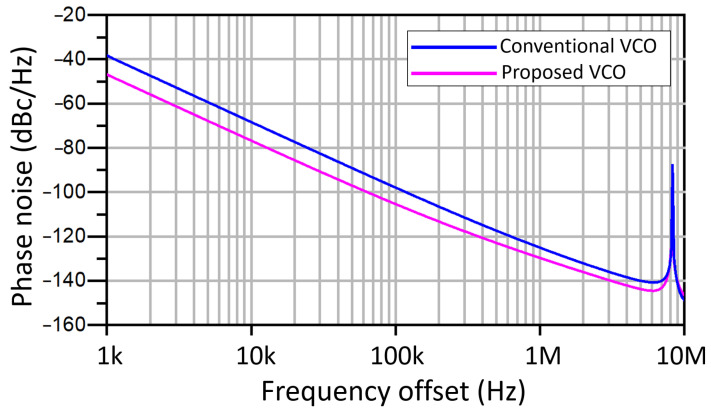
Phase noise of the conventional and proposed VCO.

Figure 9 and Figure 10 show the simulated output waveforms $V_{o1}$ and $V_{o2}$ and the tail current ($I_{tail}$) of a conventional LC-VCO and our proposal. The transistor models used are typical BSIM3 model that loosely model a 0.25 µm CMOS process. They are based on measured data from MOSIS for a 0.25 µm process and have been modified by to create the 3.3 V devices and 0.35 µm gate lengths using a thicker oxide. The models include noise and use the Advanced Compact MOSFET (ACM) model. The simulation shows that in the conventional case $I_{tail}$ is not symmetric while our proposed VCO presents a much more symmetric $I_{tail}$. Figure 11 compares the phase noise of our proposed VCO with the conventional VCO. The phase noise of our proposed VCO is −105.4 dBc/Hz at 100 kHz offset frequency, which is 7.5 dB lower than the simulated conventional VCO. Due to the parasitics introduced by M3 and M4 to the LC tank, the proposed solution lowers the output frequency. This effect is minor, but must be taken into account when using this topology.

## 4. Measurement Results

**Figure 12:**
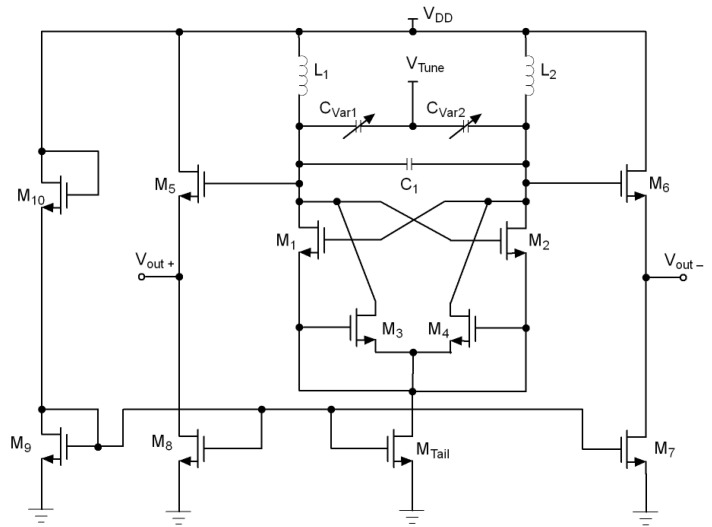
Simplified schematic of the fabricated VCO.

**Figure 13:**
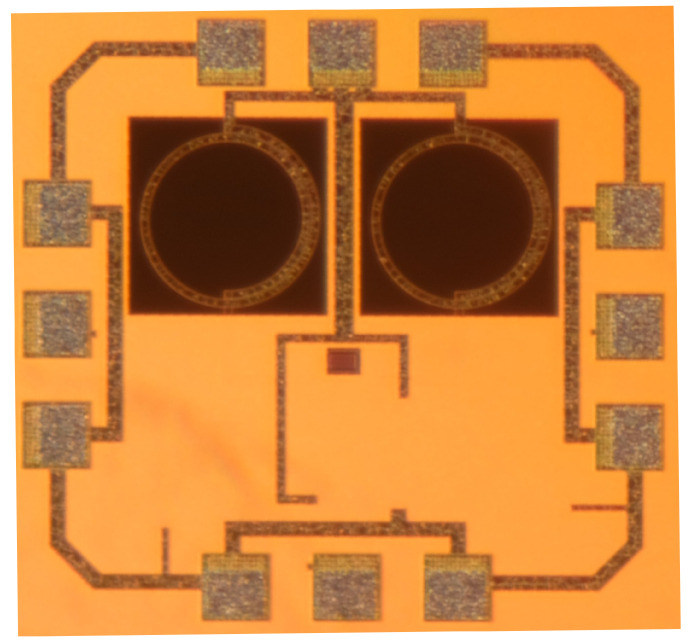
Microphotograph of the fabricated VCO.

To test the proposed topology, a prototype chip was designed using UMC 90 nm CMOS process. Figure 12 and Figure 13 shows the circuit simplified schematic and microphotograph, respectively. The oscillator outputs are buffered using CMOS source followers to provide additional isolation from load variations and to boost the output power. The tank was tuned to 1.8 GHz, comprising two 1.15 nH with 1.5 turns inductors with a Q of 14, a 3.27 pF metal-oxide-metal (MOM) capacitor and two varactors. A voltage applied to the $V_{Tune}$ pin, which is connected to varactors, controls the VCO oscillation frequency. The total area occupied by the circuit is 750 × 850 μm${}^{2}$ including the pads for on wafer measurement. Table 1 summarizes the value of the components of the VCO.

**Figure 14:**
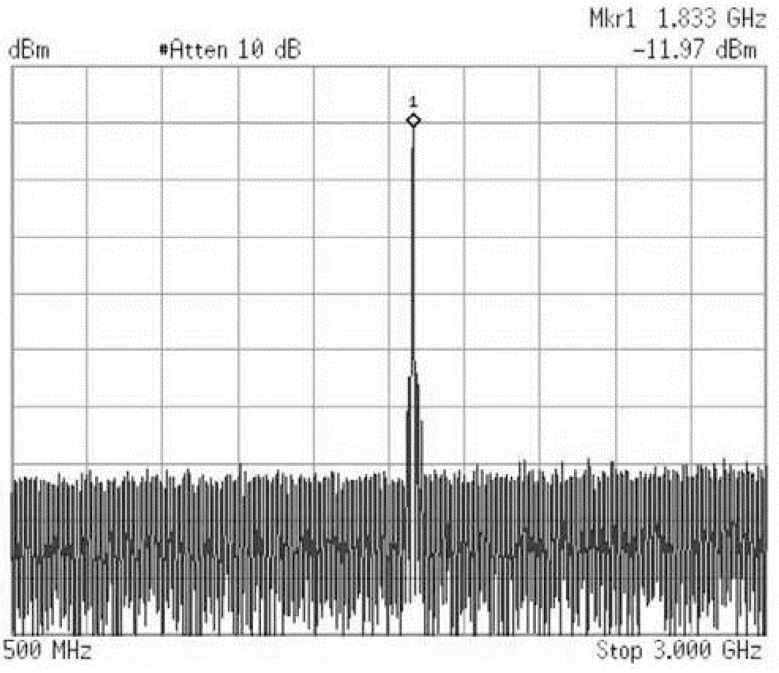
VCO Output Spectrum.

**Figure 15:**
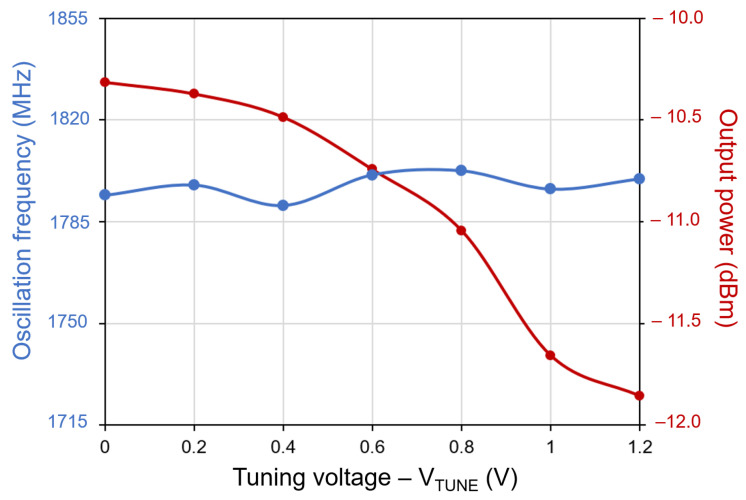
Measured frequency and output power vs. tuning voltage.

**Figure 16:**
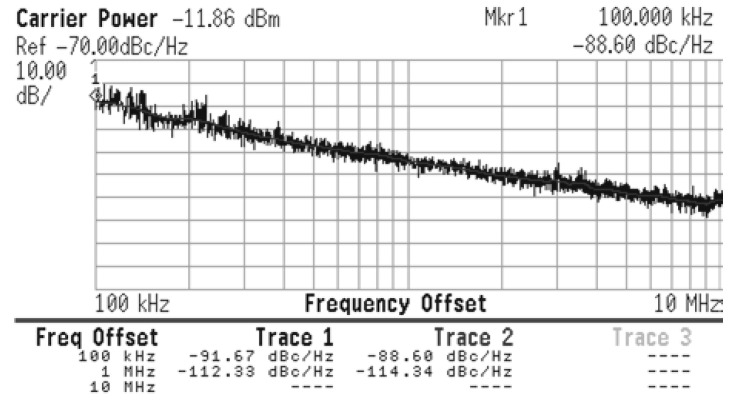
VCO measured phase noise.

Figure 14 shows the measured frequency spectrum of the proposed VCO when $V_{Tune}=0$, the output power is −12 dBm at 1.83 GHz oscillating frequency. The measured loss of the combination of the probe and cable is 1.1 dB, so the output power is −10.87 dBm. As shown in Figure 15, the output power keeps almost constant while the oscillation frequency can be tuned from 1.72 GHz to 1.83 GHz as the control voltage ranges from 1.2 to 0 V. The measured phase noise is shown in Figure 16. Table 2 compares the measured and simulated results indicating a good agreement between simulation and measurement. The VCO prototype core consumes 3.3 mA at 1.2 V supply and the total power consumption of the VCO, including the output buffers and bias, is 15.84 mW.

A brief overview of similar works available in the literature is given in Table 3. The performances of the VCOs oscillating at different frequencies are compared using the typical figure-of-merit (FoM) [33]:

(2) $$FoM\left[\mathrm{dBc}\right]=L\left(\Delta f\right)-20\;\log\left(\frac{f_{0}}{\Delta f}\right)+10\;\log\left(\frac{P_{DC}}{1\;\mathrm{mW}}\right),$$

where L(Δf) is the phase noise (PN) in dB at the frequency offset Δf, $f_{0}$ is the center oscillation frequency and $P_{DC}$ is the power consumption. FoM is specified at frequency offset of 1 MHz. As seen in Table 3, CMOS VCOs exhibit better performance than their NMOS counterparts. This is because CMOS structures provide higher transconductance for a given bias current [34]. As can be derived from Table 3, the VCO proposed in this paper compares to reported NMOS VCOs. Its main disadvantage is the operating frequency and tuning range, but this is due to the fact that we have designed the VCO for a lower frequency and tuning range. If a capacitor bank and a higher operating frequency where used, a better FoM would be achieved. The presented VCO phase noise and power consumption are comparable with the reported self-biasing NMOS VCOs. This is accomplished by adding just two transistors, and no additional electronics is required to generate the biasing. This fact is reflected in the area, which is one of the lowest reported. The area could have been further reduced if a symmetrical inductor had been used in the tank instead of two conventional inductors.

## 5. Conclusions

A new current-shaping technique to reduce VCO phase noise has been proposed. This method uses a feedback injection mechanism that only uses two transistors and reduces the phase noise as compared to the conventional designs, with a minimum penalty in area and power consumption. To test the proposed solution, simulations were performed and used for evaluation and comparison. Also, a prototype chip fabricated in a 90 nm CMOS process was used to verify the proposed solution. The oscillation frequency can be tuned from 1.72 GHz to 1.83 GHz with an output power of −12 dBm at 1.83 GHz. The power consumption of the core is 3.96 mW. The phase noise results, −112 dBc/Hz at 1 MHz offset, indicates a good agreement between simulation and measurement.

## Figures and Tables

**Table 1:** VCO componentes values.

Component	Value
M_1_ & M_2_	W_finger_ = 1 μm, L = 200 nm, Multiplicity = 20
M_3_ & M_4_	W_finger_ = 8 μm, L = 360 nm, Multiplicity = 30
M_5_ & M_6_	W_finger_ = 530 nm, L = 100 nm, Multiplicity = 8
M_7_ & M_8_	W_finger_ = 530 nm, L = 100 nm, Multiplicity = 8
M_9_	W_finger_ = 600 nm, L = 250 nm, Multiplicity = 10
M_10_	W_finger_ = 500 nm, L = 100 nm, Multiplicity = 14
C_VAR1_ & C_VAR1_	C_MAX_ = 3.824 pF
L_1_ & L_2_	L = 1.15 nH, Q = 14@2.2 GHz, 1.5 turns
C_1_	C = 1.364 pF

**Table 2:** VCO simulated and measured phase noise. Average values for 1793 and 1833 MHz.

Frequency Offset	Simulated Phase Noise	Measured Phase Noise
100 kHz	−85 dBc/Hz	−86.6 dBc/Hz
1 MHz	−111.5 dBc/Hz	−112.2 dBc/Hz
5 MHz	−130 dBc/Hz	−125.5 dBc/Hz

**Table 3:** VCO performance comparison.

Ref.	Year	Bias Scheme	Process	VCO	Supply	Freq.	Tuning	PN@1 MHz	Power	Area	*FoM*@1 MHz
			(nm)	Type	(V)	(GHz)	Range (%)	(dBc/Hz)	(mW)	(mm${}^{2}$)	(dBc/Hz)
**[35]**	2019	Self-biasing w. fixed DC	180	CMOS-LC	1.2	2.45	28.6	−120	1.73	0.938	−185
**[29]**	2019	Self-biasing w. fixed DC	180	CMOS-LC	0.8	1.4	18	−123	0.7	2.706	−187
**[36]**	2015	Self-biasing w. fixed DC	180	CMOS-LC	1.2	2.55	9.2	−123	3.2	0.332 *	−186
**[37]**	2015	Self-biasing w. fixed DC	130	CMOS-LC	1.4	2.4	1.7	−128	4.2	0.092 *	−190
**[38]**	2019	Self-biasing w. adaptative DC	180	CMOS-LC	1.2	4.55	4.3	−123	1.35	0.979	−195
**[39]**	2009	Self-biasing w. adaptative DC	130	NMOS-LC	0.6	4.85	10.2	−117	3.9	0.723	−185
**[40]**	2020	Self-biasing w. fixed DC	65	NMOS-LC	0.45	10.4	13.6	−115	2.7	0.660	−191
**[41]**	2011	Self-biasing w. fixed DC	65	NMOS-LC	1.2	20	17	−107	19.2	0.800	−180
**This work**	**2021**	**Feedback injection curr.**	**90**	**NMOS-LC**	**1.2**	**1.77**	**6.2**	**−112**	**3.96**	**0.638**	**−171**

* Dimensions excluding pads.

## Abbreviations

The following abbreviations are used in this manuscript:

ACM	Advanced Compact MOSFET
CMOS	Complementary Metal-Oxide-Semiconductor
F	Noise Factor
FoM	Figure of Merit
LC-VCO	Inductor-Capacitor Voltage Controlled Oscillator
ISF	Impulse Sensitivity Function
MOM	Metal-Oxide-Metal
PLL	Phase Locked Loop
Q	Quality Factor
VCO	Voltage-Controlled Oscillator
WSNs	Wireless Sensor Networks
